# Antiproliferative and Pro-apoptotic Activities of *Tournefortia mutabilis* vent. Leaves on the Human Breast Adenocarcinoma Cell Line (MCF-7)

**DOI:** 10.5812/ijpr-149405

**Published:** 2024-09-16

**Authors:** Zoila Mora-Guzmán, Luis Bernardo Flores-Cotera, Eduardo Pérez-Campos, Rebeca López-Marure, Delia Soto-Castro, Felipe Alonso Masso-Rojas, Araceli Paéz Arenas, Edgar Zenteno, Margarito Martinez-Cruz, Laura Pérez-Campos Mayoral, María Teresa Hernández-Huerta, María del Socorro Pina-Canseco

**Affiliations:** 1 UNAM-UABJO, Faculty of Medicine Research Center, Faculty of Medicine and Surgery, Autonomous University Benito Juarez of Oaxaca, Oaxaca, Mexico; 2 Department of Biotechnology and Bioengineering, Center for Research and Advanced Studies of the National Polytechnic Institute (Cinvestav-IPN), Oaxaca, Mexico; 3 National Institute of Technology of Mexico, Technological Institute of Oaxaca, Oaxaca, Mexico; 4 Department of Physiology, National Institute of Cardiology Ignacio Chávez, Mexico City, Mexico; 5 CONAHCYT, National Polytechnic Institute, CIIDIR Oaxaca Unit, Oaxaca, Mexico; 6 Translational Medicine Laboratory, UNAM-INC Unit, National Institute of Cardiology Ignacio Chávez, Mexico City, Mexico; 7 Department of Biochemistry, Faculty of Medicine, National Autonomous University of Mexico, Mexico City, Mexico; 8 CONAHCYT, Faculty of Medicine and Surgery, Autonomous University Benito Juarez of Oaxaca, Oaxaca, Mexico

**Keywords:** Apoptosis, Medicinal Plants, Anticancer, Pro-apoptotic, Breast Neoplasms, MCF-7 Cells

## Abstract

**Background:**

Breast cancer is the most common cancer among women worldwide, impacting not only the patients but also their families and communities. *Tournefortia mutabilis* vent. is a plant endemic to Mexico, traditionally used in Zapotec medicine for the treatment of cancer.

**Objectives:**

This study aims to evaluate the effects of the chloroformic extract of *T. mutabilis* vent. leaves on cell proliferation and cell death in MCF-7 cells.

**Methods:**

The effect of the extract on MCF-7 cell proliferation was measured using 3-(4,5-dimethylthiazol-2-yl)-2,5-diphenyltetrazolium bromide (MTT) and crystal violet staining. Apoptosis was evaluated through fluorescein diacetate/propidium iodide staining and caspase-3, -6, and -9 activity assays.

**Results:**

The half-maximal inhibitory concentration (IC50) of the *T. mutabilis *vent. extract on MCF-7 cell proliferation at 48 hours and 72 hours after treatment was 86.4 µg/mL and 2.74 µg/mL, respectively. We observed that the extract and its semi-purified fractions induced cell death through the activation of caspases 3, -6, and -9.

**Conclusions:**

*Tournefortia mutabilis *vent. is a potential source of compounds with antiproliferative and pro-apoptotic activities on the MCF-7 cell line, primarily through the intrinsic pathways of apoptosis.

## 1. Background

Cancer is the second leading cause of death worldwide, following cardiovascular diseases; however, in middle- and high-income countries, it has become the leading cause of death (1). The use of medicinal plants as a source of new compounds with potential therapeutic applications as anticancer agents is widely recognized—more than 67% of the currently approved anticancer drugs were originally discovered or derived from plants, marine organisms, or microorganisms (2). Mexico is home to more than 4,500 medicinal plant species, making it one of the most biodiverse regions globally in terms of medicinal flora (3). Despite this richness, only a small fraction of these plants, and plants worldwide, have been subjected to phytochemical research, and even fewer have been explored for their pharmacological properties (4). Indigenous communities often possess extensive knowledge of locally available medicinal plants. For instance, Frei et al. 1998 (5) studied the traditional medicine of the Zapotecs, a pre-Colombian indigenous civilization with a cultural history dating back at least 2,500 years (6). Their research focused on the botanical diversity of the state of Oaxaca, Mexico, recording 445 species, primarily those used for dermatological and gastrointestinal conditions. *Tournefortia mutabilis* vent. was among the plants documented.

*Tournefortia mutabilis *vent. belongs to the kingdom: Plantae; phylum: Tracheophyta; class: Magnoliopsida; order: Boraginales; family: Boraginaceae; subfamily: Boraginoideae; genus: *Tournefortia* [National Herbarium of Mexico (MEXU)]. Mexico is a significant center of diversity for the *Tournefortia *genus, with 26 species recorded (7). Several synonyms have been recognized for *T. mutabilis *vent., including *T. densiflora *m. Martens & Galeotti; *T. hartwegiana* Steud; *T. scholastica* standl. & L.O. Williams; *T. trichocalycina *DC; and *T. undulata* Benth (8). The plant is known by various common names, including “tlalchichinol,” “topoya,” “tlachichinule,” “tlachinol,” and “tlachinole” (9). In the central valleys and on the Oaxaca coast, it is also referred to as the cancer herb, black herb, and in the Zapotec language as “guìzh-blàg-chôg” (herb + leaf + rough) and “guìzh-cânzr” (herb + cancer) (10).

*Tournefortia mutabilis *vent. has a geographical distribution that includes Central America and Mexico. The Boraginaceae family, to which this plant belongs, is highly valued by indigenous communities, particularly for its medicinal properties. Infusions of *T. mutabilis *vent. are traditionally used for douches and washing gangrenous body parts, as well as a drink to relieve pain and as an adjuvant in cancer treatment (11)

To date, relatively few studies have been conducted on *T. mutabilis *vent.; however, some aspects of its biological activity have been evaluated. For example, it has been reported to have antibiotic properties, which may explain its use in treating superficial skin infections (11). Nevertheless, there are no available reports on the antitumor activity, especially on cancer cells, of any species belonging to the *Tournefortia *genus. Further research on this underexplored plant, which has recognized ethnobotanical medicinal value, could lead to the discovery of novel drugs for cancer treatment.

## 2. Objectives

The aim of this study was to evaluate the effects of the chloroformic extract of *Tournefortia mutabilis* vent. leaves on cell proliferation and cell death in the human breast adenocarcinoma cell line (MCF-7).

## 3. Methods

### 3.1. Collection and Authentication of Plant Material

The leaves of *T. mutabilis *vent. were collected from the site known as "La Pila" near San Pedro Juchatengo, Juquila, Oaxaca (16° 20′ 35″ N and 97° 5′ 15″ W, at 860 masl). The collected samples were deposited and authenticated (voucher number 1439220) at the National Herbarium of Mexico (MEXU) of the National Autonomous University of Mexico (UNAM).

### 3.2. Preparation of Plant Extracts

Preliminary experiments using different plant tissues (flowers, fruits, leaves, and stem) in combination with various extraction solvents (chloroform, methanol, and water) indicated that the chloroform extract prepared from the leaves of *T. mutabilis *vent. exhibited the most significant inhibition of MCF-7 cell proliferation compared to other tissue-solvent combinations (data not shown). Based on these findings, the leaves of *T. mutabilis *vent. and chloroform were selected for the extraction process in this study.

The leaves were washed twice with tap water and twice with distilled water, then drained and dried in sunlight for 14 days. The dried plant material (50 g) was ground into powder and extracted with 250 mL of reagent-grade chloroform (Merck KGaA, Darmstadt, Germany) with constant stirring for 24 hours. The chloroform extract was then filtered using Whatman #2 filter paper (Healthcare Life Sciences, Sheffield, United Kingdom). The solvent was removed under vacuum at 40°C using a Buchi^®^ R-100 rotary evaporator (BUCHI Labortechnik AG, Flawil, Switzerland), dried under a nitrogen atmosphere, and stored at 4°C until use.

### 3.3. Cell Lines and Cell Culture Maintenance

The MCF-7 cell line (from the American Type Culture Collection: ATCC HTB-22) was cultured in RPMI 1640 medium supplemented with L-glutamine (Caisson Laboratories, Inc. USDA), 10% fetal bovine serum (FBS, Mediatech Inc. USDA), and 1% antibiotic-antimycotic solution (100X, Caisson Laboratories, Inc. USDA) containing streptomycin, gentamicin, and ampicillin. The cells were incubated in a humidified atmosphere with 6% CO_2_ at 37°C in an MR Incubator (Binder, Germany).

The immortalized human dermal microvascular endothelial cell line (HMEC-1, from ATCC CRL-3243) was cultured in MCDB-131 medium (Caisson Laboratories, Inc. USDA), supplemented with 15% FBS (Mediatech Inc. USDA), 10 mM L-glutamine (Sigma-Aldrich Co., Missouri, USA), 10 ng/mL endothelial growth factor (EGF, Boehringer, Mannheim, Germany), and 1 μg/mL water-soluble hydrocortisone (Sigma-Aldrich Co., Missouri, USA). The cells were incubated in 6% CO_2_ at 37°C.

### 3.4. Cell Treatment and Proliferation Assay

Due to its relatively low aqueous solubility, the dried crude extract of *T. mutabilis *vent. was separately solubilized in chloroform, hexane, and ethanol, and then mixed with the culture medium to achieve the desired extract concentrations of 2.5, 5, 10, 20, 40, and 80 µg/mL. The solvent concentration in the reaction mixture was consistently maintained at 1% v/v. The resultant mixture was immediately used for the cell assays as described below.

MCF-7 cells were seeded in 96-well plates (Costar Corning, Inc., USA) at a density of 5,000 cells per well, cultured for 24 hours in a humidified chamber with 6% CO_2_ at 37°C, and then incubated in the reaction mixture for 12, 24, 48, and 72 hours. HMEC-1 cells were seeded in 96-well plates (Costar Corning, Inc., USA) at a density of 6,500 cells per well, cultured for 24 hours in a humidified chamber with 6% CO_2_ at 37°C, and then incubated in the reaction mixture for 48 hours.

Staurosporine (1 µM, 0.46 µg/mL), which induces apoptosis after 24 hours, was used as a positive control (Antonsson and Persson, 2009). All solvents were supplied by Merck KGaA (Darmstadt, Germany). Cell proliferation of MCF-7 and HMEC-1 cells was assessed using the 3-(4,5-dimethylthiazol-2-yl)-2,5-diphenyltetrazolium bromide (MTT) assay (AppliChem GmbH, Darmstadt, Germany) and crystal violet staining.

For the MTT assay, after treatment, 20 µL of MTT solution (5 mg/mL) was added to the cells and incubated for 4 hours at 37°C. Formazan crystals formed during this process were solubilized in acidified isopropyl alcohol (50 µL), and absorbance was recorded at 570 nm using a plate reader (Thermo Scientific, USDA).

For the crystal violet assay, after removing the supernatant, MCF-7 or HMEC-1 cells were fixed with glutaraldehyde (1.1%, 50 µL per well) and incubated for 10 minutes. Following the removal of glutaraldehyde, cells were washed with tap water, then stained with 50 µL per well of 0.1% crystal violet (prepared in 200 mM formic acid, pH 3.5) and incubated for 20 minutes. Excess crystal violet was removed with tap water, and the incorporated dye was solubilized with 10% acetic acid (50 µL per well). Absorbance was recorded at 595 nm (12).

The percentage of inhibition was calculated using the following equation:


$$\%\;inhibition=\;\frac{Aa}{Aa0}\times 100$$

Where Aa = Absorbance of treated cells and Aa = Absorbance of untreated cells (control) (13).

### 3.5. Apoptosis Assay

Apoptosis was evaluated using 2’,7’–dichlorofluorescin diacetate (DCFDA) (Sigma-Aldrich Co., Missouri, USA) and propidium iodide (PI) (Miltenyi Biotec, Bergisch Gladbach, Germany) staining. DCFDA is a doubly acetylated derivative of the green fluorescent dye fluorescein. It can easily diffuse through the phospholipid bilayer of cell membranes, and upon cleavage of the two DCFDA acetyl groups by intracellular esterases, the negatively charged fluorescein groups are retained within cells with intact membranes (13). In contrast, PI is a dye that cannot diffuse through intact membranes and is generally excluded from viable cells. However, PI can penetrate damaged membranes of dead cells and bind to DNA by intercalating between the bases, making it useful for identifying cells undergoing cell death.

In this study, DCFDA-PI double staining was used simultaneously to evaluate both dead and viable cells (14). MCF-7 cells, at a concentration of 200,000 cells/mL, were seeded in 6-well plates (Costar Corning Inc., New York, USA) containing RPMI 1640 medium and incubated for 24 hours in a humidified atmosphere containing 6% CO_2_ at 37°C. Following incubation, the spent culture medium was removed, fresh medium was added, and the cells were treated for 48 hours with the extract (86.4 µg/mL), 3% H_2_O_2_ (positive control), chloroform, or left untreated.

After treatment, the culture medium was removed, and the cells were washed with cold 1X PBS (1 mL). They were then treated with 60 µL of a staining solution (50 µL PI and 10 µL DCFDA) and incubated in the dark at room temperature for 10 minutes. Cells were subsequently analyzed at 10X, 20X, and 40X magnification using a Leica DM2000 fluorescence microscope (Leica Microsystems GmbH, Wetzlar, Germany).

### 3.6. Caspase Assays

The ApoAlert caspase fluorescent assay kit (Clontech Laboratories Inc., California, USA) was used according to the manufacturer’s instructions to assess the protease activity of caspases 3 and 9/6, which are activated at different stages and/or pathways of the apoptotic process.

MCF-7 cells, at a concentration of 200,000 cells/mL, were seeded in 6-well plates containing RPMI 1640 medium and incubated for 24 hours in a humidified atmosphere with 6% CO_2_ at 37°C. After incubation, the cells were treated with *T. mutabilis vent*. extract (86.4 µg/mL) or with fraction F8 (86.4 µg/mL), which was obtained by chromatographic fractionation of the crude extract (as described below). The treated cells were incubated for an additional 18 hours.

Following treatment, the cells were fixed with 2% paraformaldehyde for 10 minutes, washed with 1 mL PBS, and 100 µL of permeabilizing buffer diluted in injectable water was added. Next, 50 µL of 2X reaction buffer containing DTT were added to the cells, which were then incubated on ice for 30 minutes. Subsequently, 5 µL of caspase-3 substrate (50 µM) or 5 µL of caspase-9/6 substrate (250 µM) were added to the cells. The cells were then incubated for one hour in a 37 °C water bath. After incubation, nuclei were labeled with 4',6-diamidino-2-phenylindole (DAPI) (blue color). Images were captured using an LSM-700 confocal microscope (Carl Zeiss, Baden-Württemberg, Germany). Controls included cells grown in RPMI 1640 medium alone and cells grown in the medium with chloroform. Each experiment, whether with the *T. mutabilis *vent. extract, the fraction F8, or the controls, was performed in triplicate.

### 3.7. Chromatographic Fractionation of the Crude Extract

The crude extract (3 g) was fractionated by silica column chromatography (CC) using 55.5 g of silica. The elution was carried out with mixtures of hexane, ethyl acetate, and methanol with increasing polarity. The solvent percentages used were as follows: 100 - 0 - 0, 95 - 5 - 0, 90 - 10 - 0 (two volumes), 85 - 15 - 0, 80 - 20 - 0 (six volumes), 66 - 34 - 0 (four volumes), 0 - 100 - 0 (five volumes), and 0 - 0 - 100 (two volumes), where one volume corresponds to 100 f. The collected fractions were analyzed by thin-layer chromatography (TLC), and those displaying a similar band pattern were grouped into 22 new fractions.

From these 22 fractions, five (F3, F4, F8, F13, and F18) were selected for further investigation based on their TLC profiles, specifically because they exhibited the lowest number of bands (2 - 4) on the TLC plates, indicating potential purity or simplicity in composition. These five fractions were then subjected to cell proliferation inhibition assays.

After solvent removal from the fractions at 40°C under vacuum using a Buchi^®^ R-100 rotatory evaporator (BUCHI Labortechnik AG, Flawil, Switzerland), the samples were transferred to amber vials, dried under air drag for 12 hours, and stored at 4°C until use. Colorimetric phytochemical assays were conducted to identify the secondary metabolite groups present in each of the 22 fractions.

Interestingly, it was observed that all five selected fractions were completely soluble in ethanol. Therefore, each fraction was solubilized in ethanol prior to being evaluated for its effects on cell proliferation.

### 3.8. Phytochemical Screening

Qualitative assays were conducted to identify the categories of secondary metabolites present in the extract. The following tests were employed: (1) dragendorff test, for alkaloids; (2) shinoda-NaOH test, for flavonoids (15); (3) NaOH test, for coumarins (16); (4) rosenthaler-acetic anhydride test,for saponins; (5) gelatin reagent/FeCl₃ test, for tannins; (6) borntrager reaction, for quinones; (7) Baljet test, for cardiotonic glycosides; (8) hydroxylamine hydrochloride test, for sesquiterpene lactones. These tests were used to explore and identify the presence of specific secondary metabolites in the extract.

### 3.9. Statistical Analysis

All experiments were conducted in triplicate across at least three independent trials. The results are presented as mean ± standard deviation (SD). Statistical significance was determined using one-way ANOVA, with a P-value < 0.01 considered significant. The data analysis was performed using GraphPad Prism 6.00 software (La Jolla, California, US).

## 4. Results

### 4.1. Tournefortia mutabilis vent. Extract Inhibits MCF-7 Cell Proliferation

**Figure 1.**
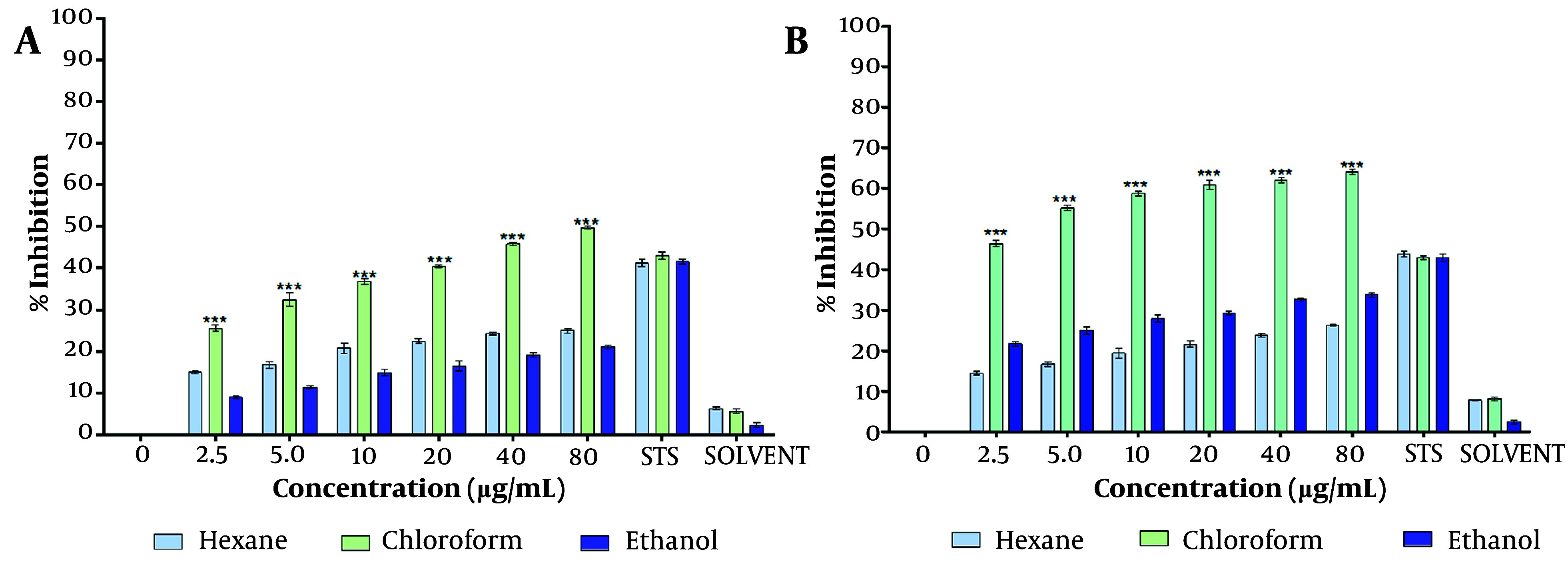
Inhibition of proliferation of MCF-7 cells treated with *Tournefortia mutabilis *vent. leaf extract. Concentrations of 4.5 to 80 ug/mL of extract were solubilized in chloroform, hexane, and ethanol, and were evaluated separately by the MTT assay. A, treatment at 48 h; and B, treatment at 72 h. The results of the experiments are expressed as percentages for untreated cells (0), and cells treated with 1 µM staurosporine (STS) were the positive control for cell death. The results shown are for one experiment representative of three independent assays (*** P < 0.001 compared to control cells).

The dried chloroform extract of *T. mutabilis* vent. leaves were resuspended in PBS (1X Phosphate Buffered Saline) and other solvents (i.e., chloroform, ethanol, hexane, ethyl acetate, and methanol) for a preliminary assessment of cell proliferation inhibition. The inhibitory effect of the *T. mutabilis *vent. extract on MCF-7 cell proliferation was evaluated using the MTT (3-[4,5-dimethylthiazol-2-yl]-2,5-diphenyl tetrazolium bromide) assay, as shown in Figure 1. 

The results indicated that the inhibition of cell proliferation was directly proportional to the extract concentration, regardless of the solvent used to resuspend the extract. However, the chloroform extract demonstrated significantly higher inhibitory activity at each concentration tested (4.5 to 80 µg/mL) compared to the extract solubilized in ethanol or hexane. Specifically, the chloroform extract exhibited an inhibition of cell proliferation ranging from 25.6% to 49.7% at 48 hours and 46.4% to 64% at 72 hours of treatment (Figure 1A, B), with the effect being directly proportional to the extract concentration.

The half-maximal inhibitory concentration (IC50) of the chloroform extract was determined to be 86.4 µg/mL and 4.7 µg/mL at 48 hours and 72 hours of treatment, respectively. Notably, at 48 hours of treatment, the percentage of cell proliferation inhibition observed with extract concentrations of ≥ 20 µg/mL was higher than that achieved with 1 µM staurosporine (0.46 µg/mL), which served as a positive control for cell death. Staurosporine is a microbial alkaloid known to induce cell death in tumor cells by causing G2/M cell cycle arrest and modulating G1 cell cycle arrest. Similarly, at 72 hours of treatment, the inhibitory activity of the extract surpassed that of staurosporine at extract concentrations of 5 µg/mL and above.

**Figure 2.**
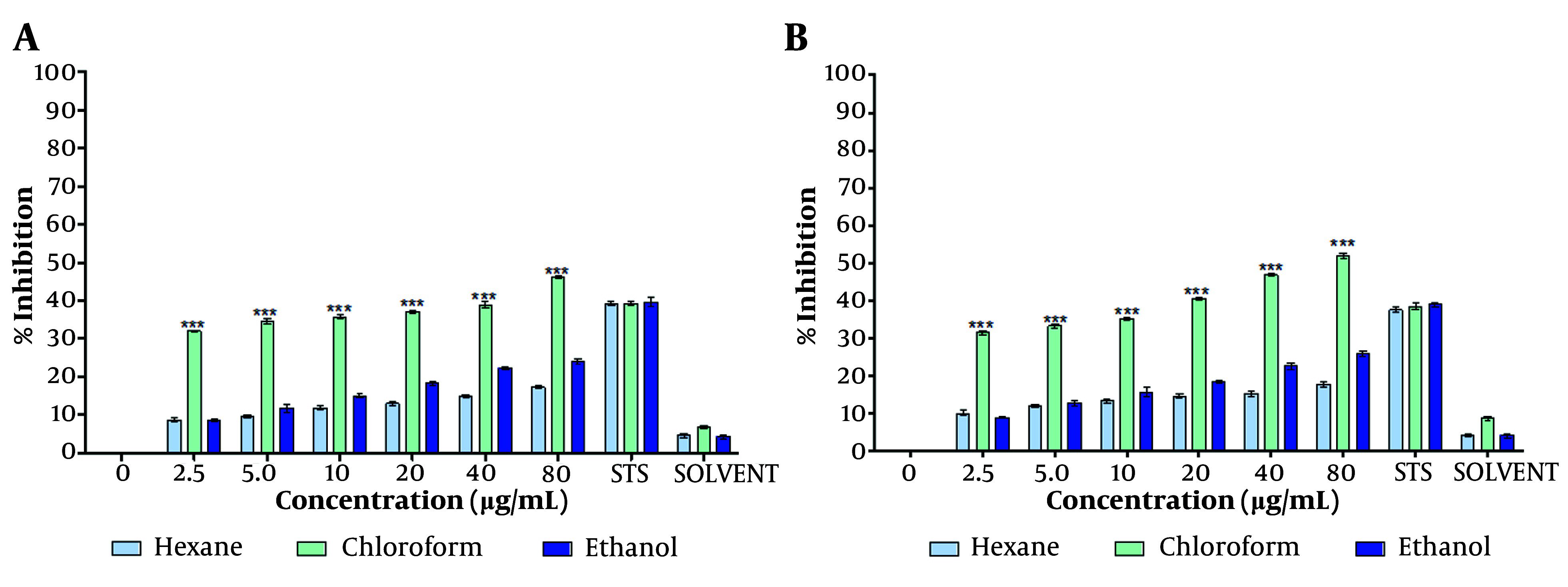
Effect of *Tournefortia mutabilis *vent. extract on MCF-7 cell proliferation determined by crystal violet assay. The extract was solubilized separately in chloroform, hexane, or ethanol. A, treatment for 48 h; and B, treatment for 72 h. The results of the experiments are expressed as percentages for untreated cells (0), and cells treated with 1 µM staurosporine (STS) were the positive control for cell death. The results shown are for one representative experiment of three independent assays (*** P < 0.001 compared to control cells).

The chloroform-dissolved crude extract significantly reduced MCF-7 cell proliferation at both 48 hours and 72 hours (Figure 1A, B) compared to the hexane- and ethanol-dissolved extracts. Consistent with the MTT assay results, the crystal violet assay further confirmed the capability of the *T. mutabilis *vent. extract to inhibit MCF-7 cell proliferation. As shown in Figure 2, an increase in proliferation inhibition was observed with increasing concentrations of the extract, regardless of the solvent used for resuspension.

Notably, the chloroform-solubilized extract exhibited much higher inhibitory activity at each concentration tested (4.5 to 80 µg/mL) compared to the extracts solubilized in either ethanol or hexane. Specifically, the inhibition of cell proliferation by the chloroform-solubilized extract varied with concentration, ranging from 34.1% to 46.0% at 48 hours (Figure 2A) and from 31.5% to 52% at 72 hours of treatment (Figure 2B). 

Overall, the ethanol-resuspended extract inhibited cell proliferation to a similar extent as the hexane-resuspended extract. However, at concentrations above 5 µg/mL, the ethanol-resuspended extract exhibited slightly higher inhibitory activity than the hexane-resuspended extract.

**Figure 3.**
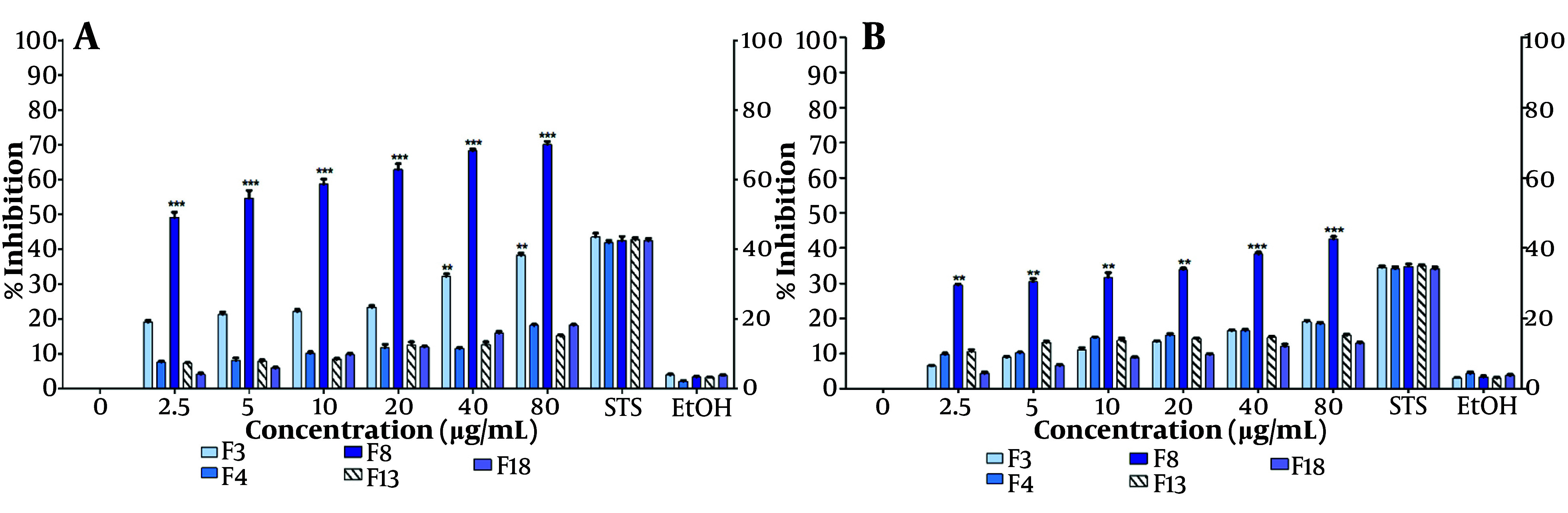
Inhibition of MCF-7 cell proliferation with fractions F3, F4, F8, F13, and F18 (at IC50 86.4 µg/mL). Fractions were obtained by chromatographic fractionation (CC) of *Tournefortia mutabilis *vent. leaf extract and solubilized in ethanol (EtOH) to evaluate: A, by MTT assay; and B, by crystal violet assay. The results of the experiments are expressed as percentages for untreated cells (0), and cells treated with 1 µM staurosporine (STS) were the positive control for cell death. The results shown are for one experiment representative of three independent assays (** P < 0.05, *** P < 0.001)

The extract was fractionated using silica CC with mixtures of hexane, ethyl acetate, and methanol of decreasing polarity. Subsequently, TLC was performed on each of the 22 fractions recovered from the CC. Five fractions, designated F3, F4, F8, F13, and F18, which had the lowest number of bands (2 - 4) on the TLC plates, were selected for cell proliferation inhibition assays after 48 hours of treatment (Figure 3A, B). Fraction F8 showed a significant inhibition of cell proliferation, ranging from 50% to 72% as assessed by the MTT assay, with the inhibition being linearly associated with the fraction concentration (Figure 3A). Notably, significant inhibition of MCF-7 cell proliferation (50%) was observed even at the lowest concentration tested (4.5 µg/mL). The pronounced inhibitory activity of fraction F8 was also confirmed at all tested concentrations using the crystal violet assay (Figure 3B). 

**Figure 4.**
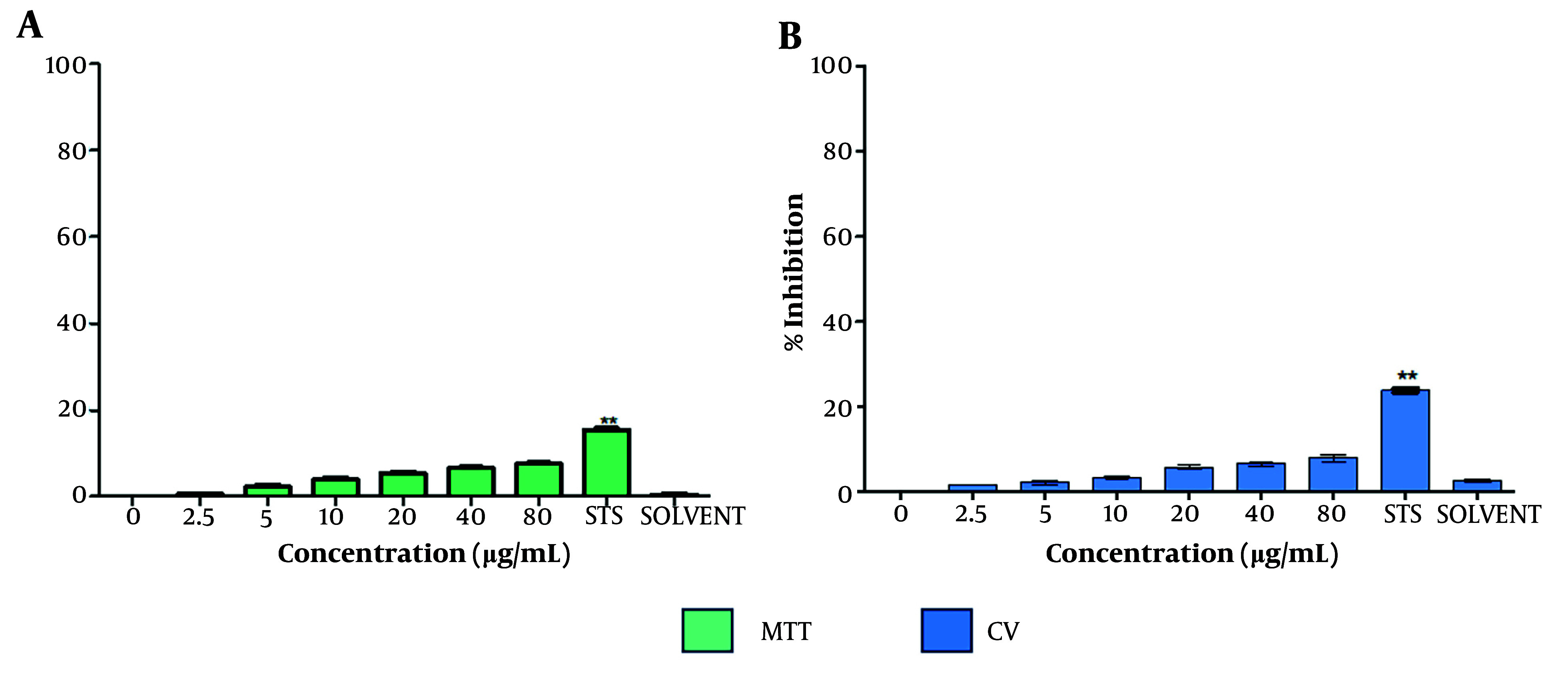
Effect of *Tournefortia mutabilis *vent. extract on HMEC-1 cell proliferation. Cells were evaluated by A, MTT; and B, crystal violet assays after 48 h of treatment. The extract was solubilized separately in chloroform (SOLVENT). The results of the experiments are expressed as percentages for untreated cells (0), and cells treated with 1 µM staurosporine (STS) were the positive control for cell death. The results shown are for one experiment representative of three independent assays (** P < 0.05 compared to control cells).

To assess the cytotoxic effect of the extract on non-cancerous cells, the inhibition of cell proliferation in HMEC-1 (human microvascular endothelial cells) treated with *T. mutabilis *vent. extract was evaluated. The results showed that *T. mutabilis *vent. extract did not exhibit cytotoxicity towards HMEC-1 cells, regardless of whether the MTT or crystal violet assay was employed, as shown in Figure 4. Specifically, the inhibition of cell proliferation was consistently 8% or less, even at the highest extract concentration of 80 µg/mL.

### 4.2. Tournefortia mutabilis vent. Extract Induces Apoptosis in MCF-7 Cells

**Figure 5.**
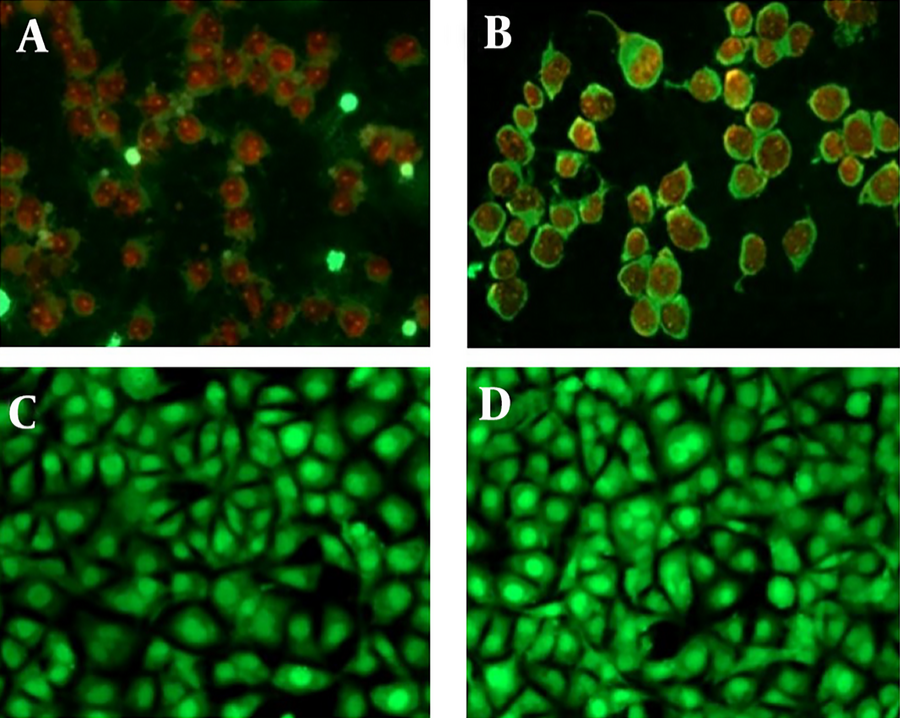
Apoptosis induced in MCF-7 cells treated with the extract of *Tournefortia mutabilis *vent. A, MCF-7 cells treated with the extract (86.4 µg/mL) solubilized in chloroform; B, MCF-7 cells treated with H_2_O_2_ (3%, 5 min.); C, Untreated MCF-7 cells were used as control; D, MCF-7 cells grown in RPMI 1640 medium treated with chloroform only. All images were taken at 20X using a Leica DM2000 microscope (Wetzlar, Germany).

We evaluated the induction of apoptosis in MCF-7 cells treated with *T. mutabilis *vent. extract using fluorescence microscopy. Figure 5A displays MCF-7 cells treated with *T. mutabilis *vent. extract for 48 hours, where the red fluorescence observed indicates cells with damaged nuclei. This damage allows the intercalation of PI with DNA, as demonstrated in the fluorescein-propidium iodide diacetate (DCFDA-IP) assay. For comparison, Figure 5B shows cells treated with hydrogen peroxide (3% for 5 minutes), a known inducer of apoptosis, exhibiting a similar effect to that of the crude extract. Untreated MCF-7 cells were used as a control (Figure 5C), along with cells treated with chloroform (Figure 5D), which showed no visible nuclear damage.

### 4.3. Tournefortia mutabilis vent. Extract and Fraction F8 Induce Caspase 3- and Caspases 9/6-Dependent Apoptosis

**Figure 6.**
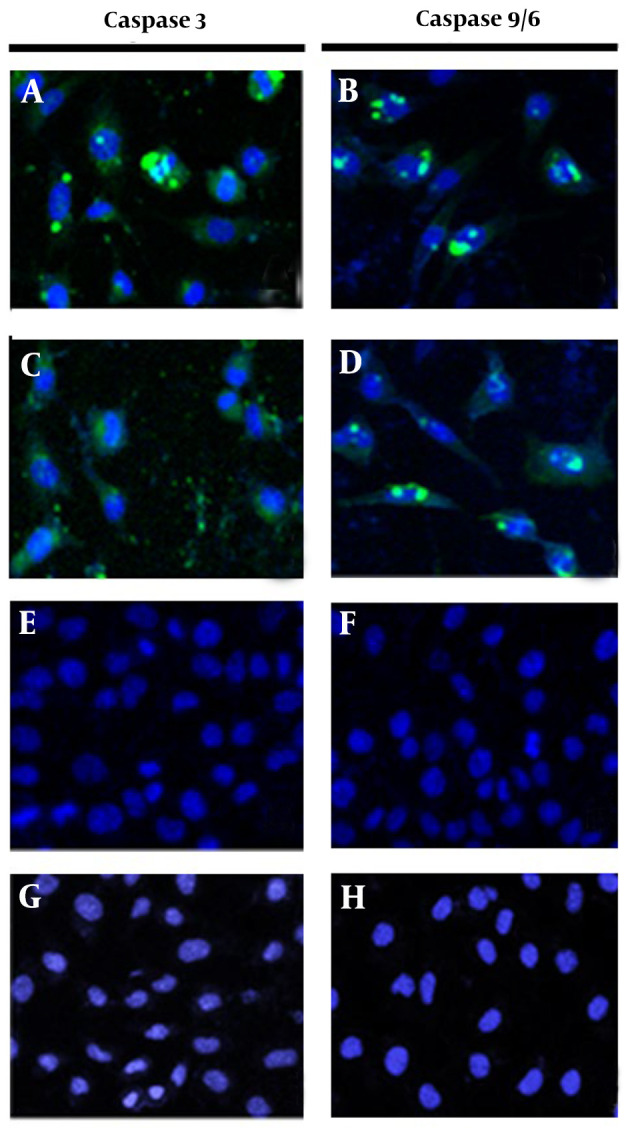
Activation of caspases 3 and 9/6. The activation of caspases 3 and 9 was evaluated in MCF-7 cells treated with 86.4 µg/mL of *Tournefortia mutabilis *vent. extract at 18 h (A and B), cells treated with fraction F8 at 86.4 µg/mL of the extract (C and D), untreated cells (E and F); and chloroform-treated cells (G and H). Images were recorded at 20X using a Carl Zeiss confocal microscope (Württemberg, Germany).

To confirm that the previously observed apoptosis was induced through caspase activation, MCF-7 cells were treated separately with the crude leaf extract of *T. mutabilis *vent. and fraction F8 for 18 hours and subsequently analyzed. Figure 6A, B display green fluorescence surrounding the cellular nucleus (with nuclei stained blue), indicating the activation of caspases 3 and 9/6, respectively. Likewise, Figure 6C, D show green fluorescence around the nucleus of MCF-7 cells treated with fraction F8, obtained through chromatographic fractionation of the extract, also indicating the activation of caspases 3 and 9/6, respectively. These findings suggest that both the crude extract and fraction F8 induce apoptosis via the activation of caspases 3 and 9/6. In contrast, Figure 6E, F (control 1, cells grown in RPMI medium) and Figure 6G, H (control 2, cells treated with chloroform) show intact cells without visible damage, confirming that none of the control conditions caused cell death.

### 4.4. Phytochemical Screening

Qualitative analysis of the leaf extract of *T. mutabilis* vent. using colorimetric phytochemical screening indicated the presence of flavonoids, tannins, quinones, terpenes, and saponins. Interestingly, in fraction F8, only flavonoids and saponins were detected. Further research is necessary to isolate and identify the specific compound(s) responsible for the observed antiproliferative and pro-apoptotic activities in *T. mutabilis *vent.

## 5. Discussion

The use of plants for the treatment of diseases has increased in recent years (17). The extraction and evaluation of various compounds from plants are common approaches in cancer research. This study is the first to scientifically investigate the anticancer activity of *T. mutabilis *vent. The initial results are consistent with the traditional medicinal use of this plant in different cultures. Our findings demonstrate that the *T. mutabilis *vent. extract inhibited the proliferation of MCF-7 cells, as measured by both the MTT assay and the crystal violet assay. Additionally, we observed the activation of caspases in MCF-7 cells treated with the extract. The inhibition of cell proliferation was significantly greater when the extract was solubilized in chloroform, with inhibition values being double or higher than those observed with hexane or ethanol (Figures 1 and 2). Similar effects of chloroform extracts have been reported in studies using the peel of *Citrus unshiu* Markovich, where apoptosis is induced in HeLa human cervical cancer cells (18). In this study, the chloroform extract of *T. mutabilis *vent. showed an IC50 of 86.4 µg/mL at 48 hours and 2.7 µg/mL at 72 hours. The damage caused by the extract to MCF-7 cells affects both the mitochondria and the cytoplasm.

The DCFDA-PI assay exclusively stains the cytoplasm of living cells, while PI produces red fluorescence when intercalated with the DNA of cells with damaged nuclei (i.e., dead cells). We also performed assays for caspases 3 and 9/6 (at 18 h, 86.4 µg/mL) to confirm that the apoptosis induced by the *T. mutabilis *vent. extract and fraction F8 occurs through the activation of these proteins. Caspase-3 is involved in the execution phase of apoptosis, leading to DNA fragmentation, chromatin condensation, and apoptotic body formation. In contrast, caspase-9, an upstream caspase in the CD95 apoptotic pathway, is activated via the mitochondrial release of cytochrome c into the cytosol (19). Activation of caspase 3 and caspase 9/6 has also been observed in studies with other extracts, such as *Fragaria ananassa* (Strawberry), which induces apoptosis in T-47D breast cancer cells (20). Our results corroborate that *T. mutabilis *vent. extract and fraction F8 induce cell death via intrinsic apoptosis, a process involving the permeabilization of the mitochondrial outer membrane, leading to apoptosome formation and the activation of caspase 9 and other effector caspases. This active caspase 9 can initiate a protease cascade that also activates caspase-3 and caspase 7. Effector caspases are responsible for initiating the degradation phases characteristic of apoptosis, including DNA fragmentation, cell shrinkage, and membrane blebbing (21). We have compared this study with other plant extracts where anti-proliferative activity in MCF-7 cells has been evaluated, as shown in Table 1. The data indicate that the extract of *T. mutabilis *vent. and fraction F8 studied here compare favorably in their ability to inhibit the proliferation of MCF-7 cells when compared to extracts from other plants, based on treatment times and IC50 (µg/mL).

**Table 1.** Antiproliferative Activity of Extracts from Different Plants on MCF-7 Cells

Plants	Study	IC50 (µg/mL)	Reference
***Tournefortia mutabilis*** ** vent.**	The chloroform extract inhibits cell proliferation after 72 h of treatment and induces apoptosis after 48 h.	2.7; 86.4	This work
***Macrosalen parasiticus***	The chloroform-water extract elicits the formation of apoptotic bodies and apoptosis at 48 h.	97.3	(22)
***Rumex thyrsiflorus*** ** fingerh**	The chloroform extract of roots inhibits 88.7% of cell proliferation after 72 h.	30	(23)
***Scrophularia variegate***	The ethanolic extract stops the cell cycle (G2/M phase) and triggers apoptosis after 48 h of treatment.	200	(24)
***Fallopia adans***	The ethanolic extract of flowers inhibits cell proliferation. The hydroalcoholic extract of flowers inhibits cell proliferation.	125; 100	(25)
***Descurainia sophia***	Aqueous extracts of seeds and aerial parts show cytotoxic activity at 48 h.	≥ 100	(26)
***Saraca indica ***	Antiproliferative activity at 72 h	73.3	(27)
***Piper cubeba***	The methanolic extract of seeds inhibits cell proliferation at 72 h.	22.3	(28)
***Hemidesmus indicus***	The hydroalcoholic extract from roots shows antiproliferative activity at 72 h.	209.7	(29)

Some indigenous medicinal plants of the Boraginaceae family have been used worldwide to treat breast cancer. For instance, the cytotoxic activity of various root extracts of *Onosma visianii* clem. was evaluated on the MDA-MB-231 breast cancer cell line. The acetone, chloroform, and ethyl acetate extracts demonstrated strong cytotoxic activity, apoptosis induction, and cell cycle arrest in the G2/M phase, with the chloroform extract showing an IC50 of 32.8 μg/mL in MDA-MB-231 cells after 24 hours of treatment (30).

Ceramella et al. examined the antioxidant properties and anti-tumor activity of the methanolic extract of aerial parts of *Anchusa azurea *Mill. using the MCF-7 cell line, which expresses estrogen, progesterone, and glucocorticoid receptors. The study reported the inhibition of MCF-7 cell proliferation with an IC50 of 524.7 µg/mL after 72 hours of treatment, along with apoptosis induction via caspases 3/7 and 9 (31). This value is considerably higher than the results observed in our study.

Shahat et al. evaluated the antioxidant and anticancer potential of *Gastrocotyle hispida*, a plant traditionally used as a diuretic and for treating rheumatism, using different cell lines. In this plant, rosmarinic acid was isolated and identified from the aerial parts and exhibited potent anticancer activity against MCF-7 cells, with an IC50 of 4.2 μg/mL after 48 hours of treatment (32). Furthermore, alcoholic extracts with an IC50 lower than 100 μg/mL are considered potential active ingredients (33).

According to these figures, the extract of *T. mutabilis *vent. shows a cytotoxicity indicator for the MCF-7 cell line, with an IC50 of less than 100 μg/mL. Additionally, the presence of flavonoids, tannins, quinones, terpenes, and saponins in the leaf extract of *T. mutabilis *vent. underscores its potential anticancer properties. Flavonoids and tannins are polyphenolic compounds known to interfere with proteins in cancer cells and induce apoptosis through DNA degradation. Many purified flavonoids have demonstrated cytotoxicity on MCF-7 cells (34). Tannic acid, a gallotannin found in several natural sources, plays a role in various cancer signaling pathways, including the JAK/STAT, RAS/RAF/mTOR, TGF-β1/TGF-β1R axis, VEGF/VEGFR, and CXCL12/CXCR4 axes (35). Saponins are steroidal glycosides with diverse activities in cancer, including cell cycle arrest, antioxidant activity, inhibition of cellular invasion, induction of apoptosis, and autophagy.

As with other phytochemical studies, the limitations include solvent concentration, temperature, and pH value, among others. Not all medicinal plant extracts contain active compounds useful for treatment. However, further studies of *Tournefortia mutabilis *vent. extract is necessary due to its demonstrated antiproliferative and pro-apoptotic activities in the MCF-7 cell line.

### 5.1. Conclusions

We found that both the chloroform extract of *T. mutabilis *vent. leaves and the fraction F8, obtained by chromatographic fractionation of the extract, significantly inhibit the proliferation of MCF-7 cells. The inhibition of cell proliferation was positively correlated with the concentration of the extract and the concentration of fraction F8, respectively. Additionally, both the chloroform extract and fraction F8 at an IC50 of 86.4 µg/mL promote apoptotic cell death in MCF-7 cells. These findings suggest that *T. mutabilis *vent. is a promising source of compounds with antiproliferative and pro-apoptotic activities against MCF-7 cells, with the effects mediated through intrinsic apoptotic pathways via the activation of caspases 3 and 9/6. This work lays the groundwork for future research aimed at identifying potentially active anti-cancer compounds from *T. mutabilis *Vent.

## Data Availability

The dataset presented in the study is available on request from the corresponding authors during submission or after publication.
